# Pure-Water-Fed Forward-Bias Bipolar Membrane CO_2_ Electrolyzer

**DOI:** 10.1021/acsami.4c02799

**Published:** 2024-05-07

**Authors:** Matthias Heßelmann, Jason Keonhag Lee, Sudong Chae, Andrew Tricker, Robert Gregor Keller, Matthias Wessling, Ji Su, Douglas Kushner, Adam Z. Weber, Xiong Peng

**Affiliations:** † Energy Technologies Area, Lawrence Berkeley National Laboratory, Berkeley, California 94720, United States; ‡ Chemical Process Engineering, RWTH Aachen University, Forckenbeckstr. 51, 52074 Aachen, Germany; § DWI Leibniz-Institute for Interactive Materials, Forckenbeckstr. 50, 52074 Aachen, Germany

**Keywords:** electrochemical CO_2_ reduction, water-fed, forward-bias, asymmetric, bipolar membrane, cell design

## Abstract

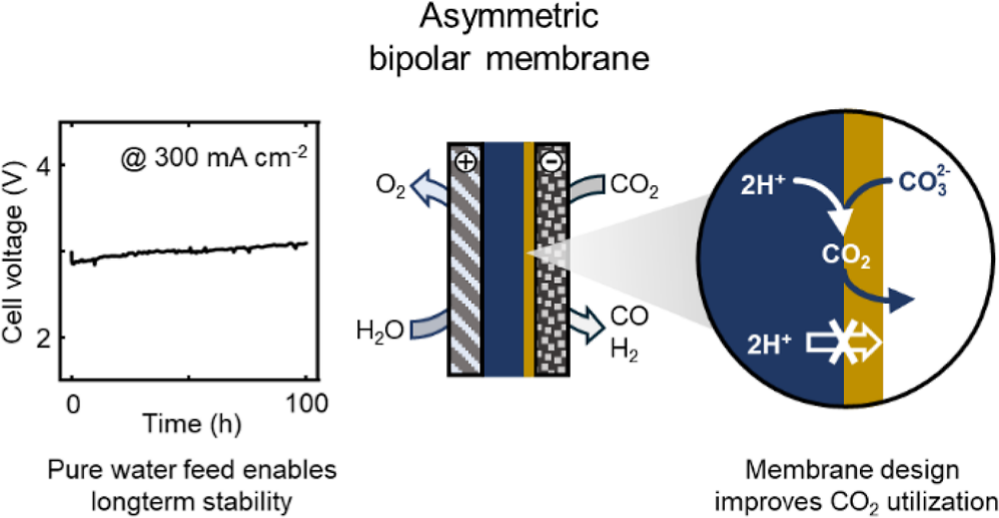

Coupling renewable electricity to reduce carbon dioxide (CO_2_) electrochemically into carbon feedstocks offers a promising pathway to produce chemical fuels sustainably. While there has been success in developing materials and theory for CO_2_ reduction, the widespread deployment of CO_2_ electrolyzers has been hindered by challenges in the reactor design and operational stability due to CO_2_ crossover and (bi)carbonate salt precipitation. Herein, we design asymmetrical bipolar membranes assembled into a zero-gap CO_2_ electrolyzer fed with pure water, solving both challenges. By investigating and optimizing the anion-exchange-layer thickness, cathode differential pressure, and cell temperature, the forward-bias bipolar membrane CO_2_ electrolyzer achieves a CO faradic efficiency over 80% with a partial current density over 200 mA cm^–2^ at less than 3.0 V with negligible CO_2_ crossover. In addition, this electrolyzer achieves 0.61 and 2.1 mV h^–1^ decay rates at 150 and 300 mA cm^–2^ for 200 and 100 h, respectively. Postmortem analysis indicates that the deterioration of catalyst/polymer–electrolyte interfaces resulted from catalyst structural change, and ionomer degradation at reductive potential shows the decay mechanism. All these results point to the future research direction and show a promising pathway to deploy CO_2_ electrolyzers at scale for industrial applications.

## Introduction

In the quest for sustainable energy systems, electrochemical CO_2_ reduction (e-CO_2_R) has emerged as a promising approach to generate carbon-containing molecules from renewable, abundant resources and not from fossil fuels. e-CO_2_R offers the potential to both mitigate greenhouse-gas emissions and produce sustainable feedstocks by coupling with renewable electricity.^1^ Despite the tremendous progress that has been made in the field of CO_2_ electrolysis, including catalyst design,^2−4^ polymer–electrolyte development,^5−7^ reaction-environment control,^8^ and fundamental insights into the cation effects,^9,10^ several challenges and limitations persist. These challenges hinder the widespread deployment e-CO_2_R as a scalable and efficient process and are attributed to issues of reactor design, mass-transport limitations, and efficient management of the reactants, all of which impact the overall efficiency and productivity.^11−13^

As high alkalinity is often desired to facilitate e-CO_2_R reactivity and selectivity,^14,15^ flow cells or zero-gap membrane-electrode assembly (MEA) with alkaline-based liquid electrolytes is used to demonstrate outstanding e-CO_2_R performance and durability.^16^ However, this leads to two major challenges. First is the formation of (bi)carbonates (CO_3_^2–^/HCO_3_^–^), due to the favorable and fast reaction of CO_2_ with hydroxide (OH^–^), which migrate from cathode to anode and subsequent protonation releases CO_2_ at the anode.^12^ This so-called CO_2_ crossover effect results in either loss of reactants or additional energy and cost penalty of regenerating and purifying CO_2_.^12,17^ Second is that cations transfer through the membrane and build up in the cathode and result in carbonate salt precipitation, which leads to blockage of CO_2_ transport pathways and resultant efficiency loss and device failure.^18,19^ Therefore, research needs to resolve both challenges of CO_2_ crossover and salt precipitation before e-CO_2_R technology can be deployed at scale for sustainable chemical and fuel productions.^20^

To address these challenges, research interest has been shifting away from conventional alkaline-based systems and looking for alternatives. For instance, although strong acidic condition is proposed in aqueous conditions for e-CO_2_R to suppress CO_2_ crossover,^21,22^ the flow-cell design requires supporting catholytes between the membrane and the cathode, which could lead to additional ohmic voltage losses.^23^ In addition, using buffering electrolytes on the cathode side can lead to operation instabilities attributed to bubble formation and liquid breakthrough.^24,25^ Other strategies such as oscillating voltage operation,^26^ CO_2_ recovery,^27^ and novel reactor design^28^ have shown promises; however, most systems still cannot operate at industrially relevant conditions or address both of the two above-mentioned challenges. More recent works report water-fed bipolar-based CO_2_ electrolyzers, which show a great promise in preventing CO_2_ crossover.^29−31^ However, these systems still face challenges in high cell voltages or periodic activation to sustain continuous and stable operation.

**Figure 1:**
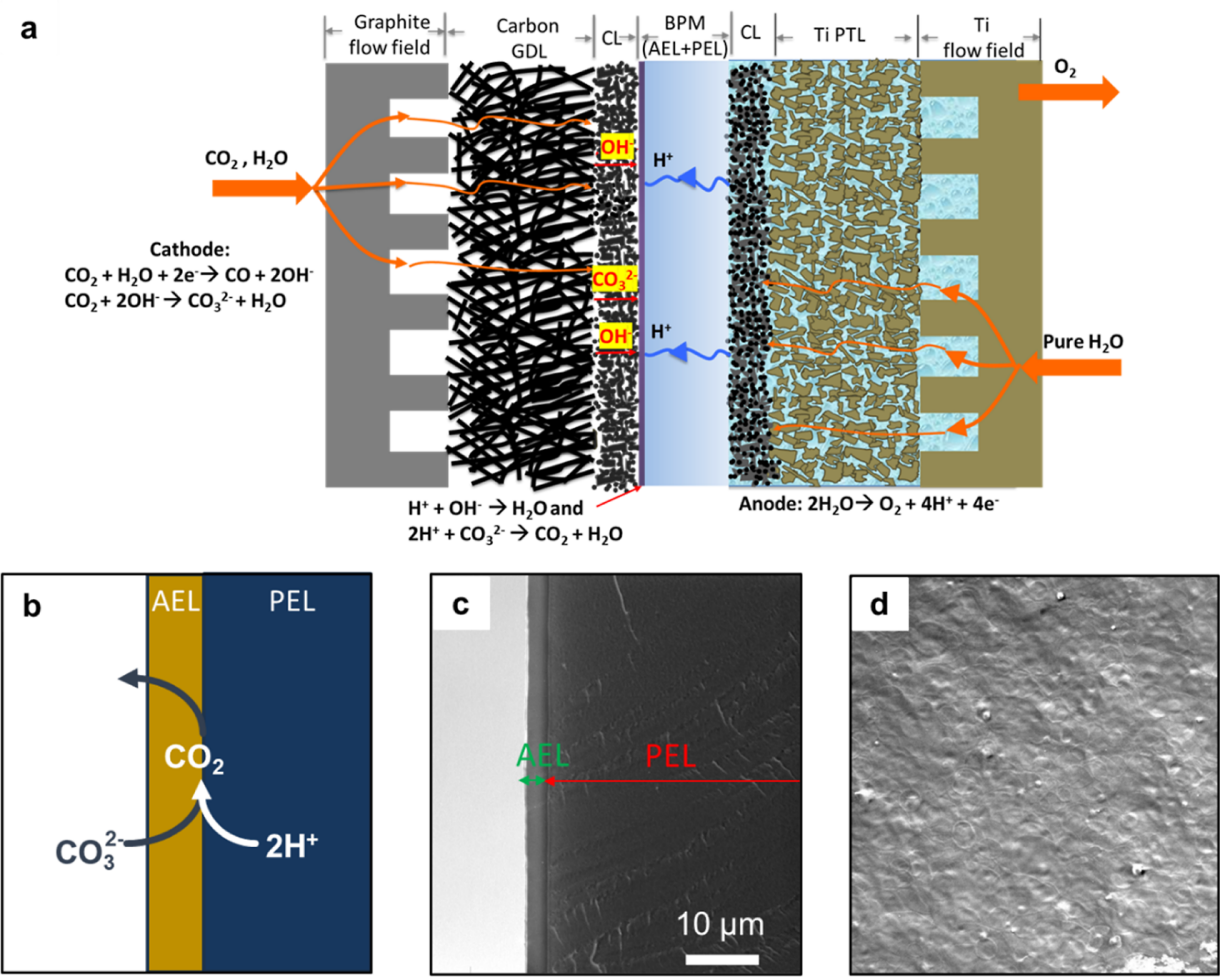
Pure-water-fed BPM CO_2_ electrolyzer concept and morphological features of the asymmetric BPM. (a) Schematic illustration of the pure-water-fed forward-bias BPM CO_2_ electrolyzer. (b) Schematic illustration of the mass-transport pathways in the bipolar junction of the asymmetric BPM. (c) Cross-sectional scanning electron microscopy (SEM) images of the BPM showing an asymmetric configuration of thin AEL and thick PEL. (d) Surface morphology of the BPM (AEL side).

Here, we propose a pure-water-fed CO_2_ electrolyzer employing a free-standing asymmetric bipolar membrane (BPM) to overcome the above-mentioned CO_2_ crossover and salt precipitation challenges commonly seen in conventional alkaline–electrolyte-based devices. The BPM CO_2_ electrolyzer in our study is operated under a forward-bias mode in a zero-gap configuration, in which CO_2_ is reduced at the cathode and water is oxidized at the anode (Figure 1a). The asymmetric BPM has a thick proton-exchange layer (PEL) made of a commercial Nafion 117 (177.8 μm) proton-exchange membrane (PEM) and a thin anion-exchange layer (AEL) fabricated by coating an anion-exchange ionomer (AEI: PiperION A) onto the PEL, as shown in Figure 1b. The coated AEL exhibits a smooth interfacial junction with the PEL and a dense surface morphology free of cracks (Figure 1c), which effectively maintains an alkaline environment at the cathode side suitable for e-CO_2_R, while the PEL blocks carbonate/bicarbonate crossover.^32^ At the bipolar junction, the recombination reaction of carbonate/bicarbonate and protons occurs and regenerates CO_2_, preferentially permeating back to the cathode side. The asymmetrical design with thin AEL allows robust CO_2_ gas back-transport from where it is generated in the junction and can possibly avoid the risk of delamination between AEL and PEL.^33^ Compared to the conventional reverse-bias BPM CO_2_ electrolyzer,^28^ the forward-bias BPM eliminates the kinetically sluggish water-dissociation reaction, which often requires specific catalysts and possible high transmembrane overpotential. Feeding the electrolyzer with pure water (deionized [DI] water: 18.2 MΩ cm) guarantees the absence of supporting electrolytes and other ions and, therefore, avoids the risk of salt precipitation. Moreover, the zero-gap cell design minimizes internal resistance and is beneficial for improving the device energy efficiency (EE). We demonstrate the robustness of the BPM CO_2_ electrolyzer using commercially available cell components with CO_2_R to CO.

## Results and Discussion

### Configuration of BPM and Impact of AEL Thickness

To understand how BPM fabrications can impact the performance of the CO_2_ electrolyzer, we compared two different configurations where the thin AEL was coated on either the cathode gas-diffusion electrode (GDE) or the PEL side. We first evaluated the AEI-coated GDE for e-CO_2_R (Figure S1), which showed relatively low CO Faradaic efficiency (FE) and substantial hydrogen-evolution reaction (HER) at high cell potentials. The AEI-coated GDE shows severe surface cracks, as indicated by the SEM images in Figure S2a,b. These cracks are likely to be formed due to coating AEI to the cathode surface, which has high surface roughness due to the imperfectness of the catalyst layer.^34^ These cracks can jeopardize the bipolar interface, as direct contact between the GDE and PEL could occur at the cracks, leading to pronounced HER. Even though the previous study argues that GDEs with cracked surfaces are expected to be more stable due to improved drainage of salt precipitates,^35^ we do not expect such benefit as no supporting electrolytes are used herein. Rather, the integrity of the bipolar junction sustained by intimate interfacial contact between the AEL and the PEL is critical. Therefore, we instead coat AEI to PEL to form free-standing BPMs for the following studies.

**Figure 2:**
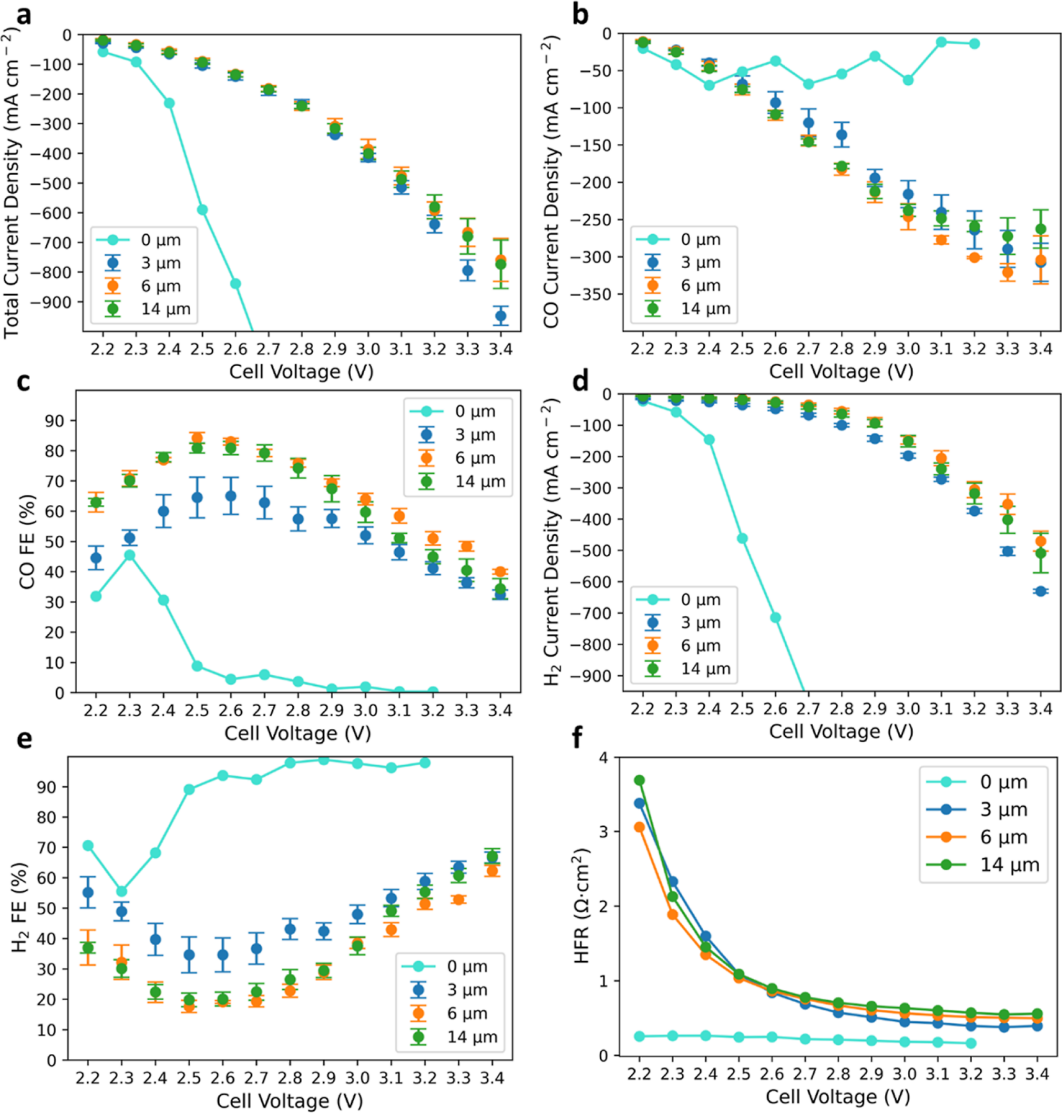
Investigations on e-CO_2_R performance with various AEL thicknesses. Dependence of (a) total current density, (b) measured CO partial current density, (c) distribution of CO FE, (d) measured H_2_ partial current density, (e) distribution of H_2_ FE as a function of applied voltage, and (f) measured high-frequency resistance (HFR) as a function of applied voltage. Operating conditions were set to a cell temperature of 60 °C, a differential pressure of 60 psi applied only on the cathode, a CO_2_ gas flow rate of 100 sccm, and a DI water flow rate of 100 mL min^–1^ on the anode. Cathode: 1.3 ± 0.1 mg_Ag_ cm^–2^ and anode: 0.2 ± 0.05 mg_Ir_ cm^–2^. The error bars represent the standard deviation of independent measurements from three identical MEAs.

To investigate how the thickness of AEL impacts the efficacy of the bipolar interface and the trade-off between overall ohmic resistance and ion crossover, we fabricated BPMs with three typical AEL thicknesses (Figure S3) of 3 ± 0.6, 6 ± 1.2, and 14 ± 2.8 μm as well as with no AEL coating (0 μm). Without the AEL coated on the PEL, it shows the highest total currents (Figure 2a) due to a significantly lower HFR (Figure 2f), as well as enhanced CO partial current density at low cell voltages (≤2.4 V, Figure 2b). However, CO FE drops significantly when increasing the cell voltage above 2.4 V, as shown in Figure 2c, reaching almost zero at voltages higher than 3.0 V. This indicates that even though AEI is used as the polymer electrolyte on the cathode catalyst layer, it cannot maintain a strong alkaline environment as protons from the anode can possibly acidify the cathode/PEM interfaces at high currents; therefore, HER dominates the cathode reaction. Applying an AEL coating as thin as 3 ± 0.6 μm can create an effective barrier for proton crossover, thus leading to enhanced CO partial current density and FE (Figure 2b,c). As the AEL thickness increases, the HER can be further inhibited (Figure 2d,e); however, the HER cannot be fully suppressed even when increasing the AEL thickness to 14 ± 2.8 μm. This indicates that the HER induced by proton crossover is not the only factor that leads to CO FE loss. The plateauing behavior of CO partial current density indicates that CO_2_ mass transport limits e-CO_2_R performance, especially at a higher applied voltage (>3.0 V). In our BPM CO_2_ electrolyzer, the electrochemically active reacting interface primarily exists at the catalyst/polymer–electrolyte interface, which could complicate the species transport.^36^

On the other hand, increasing the AEL thickness leads to a proportional increase in the ohmic resistance of the cell, especially at cell voltages above 2.6 V, as indicated by the HFR (Figure 2f), which mainly represents the ionic transport resistance through the BPM. It is also worth noting that the HFR decreases with the increase of operating voltage for all three AEL thicknesses. The descending trend in the HFR could result from a change in the charge-carrying ions through AELs. Although She et al. argue that no carbonate is formed even on the cathode and therefore OH^–^ is the only charge carrier in a similar pure-water-fed bipolar CO_2_ electrolyzer supported by isotope labeling experiments, the study cannot rule out the possibility that the released CO_2_ from recombination of carbonate and proton gets converted into products through e-CO_2_R, as the regenerated CO_2_ is located in close proximity to the cathode.^30^ As shown by Larrazábal,^13^ CO_3_^2–^ is the main charge carrier at low- to moderate-current densities, whereas OH^–^ becomes the main charge carrier at high-current densities. Due to the higher mobility of OH^–^ compared to that of CO_3_^2–^ in the AEL, the HFR decreases when the charger carrier changes at elevated potentials. The dependence of the ohmic resistance on the AEL thickness also supports the polarization behavior in Figure 2a, showing an increase in the total current density for thinner coatings at the same voltage. However, the current increase for the thin AEL coating (3 ± 0.6 μm) is mainly attributed to an increase in the H_2_ current density (Figure 2d), which leads to increased H_2_ FE (Figure 2e). Consequently, the 6 ± 1.2 μm thick AEL coating was chosen for further studies in the rest of work, thanks to the optimal balance between preventing proton crossover and reducing ohmic resistance.

As indicated in previous studies,^29,31^ one possible failure mode of the forward-bias BPM CO_2_ electrolyzer is the delamination issue between the AEL and PEL, which is projected to result in increase of contact resistance. However, this behavior is not observed for the asymmetrical BPMs in this study, as shown by the descending trend and plateauing behavior of the HFR, which further illustrates the superiority of asymmetrical design of BPM with thinner AEL to allow for efficient CO_2_ back diffusion from the bipolar junction to the cathode and possible improved heat and water management at the bipolar junction.^37,38^

### Investigating How Cathode Differential Pressure Impacts Electrolyzer Efficiencies

**Figure 3:**
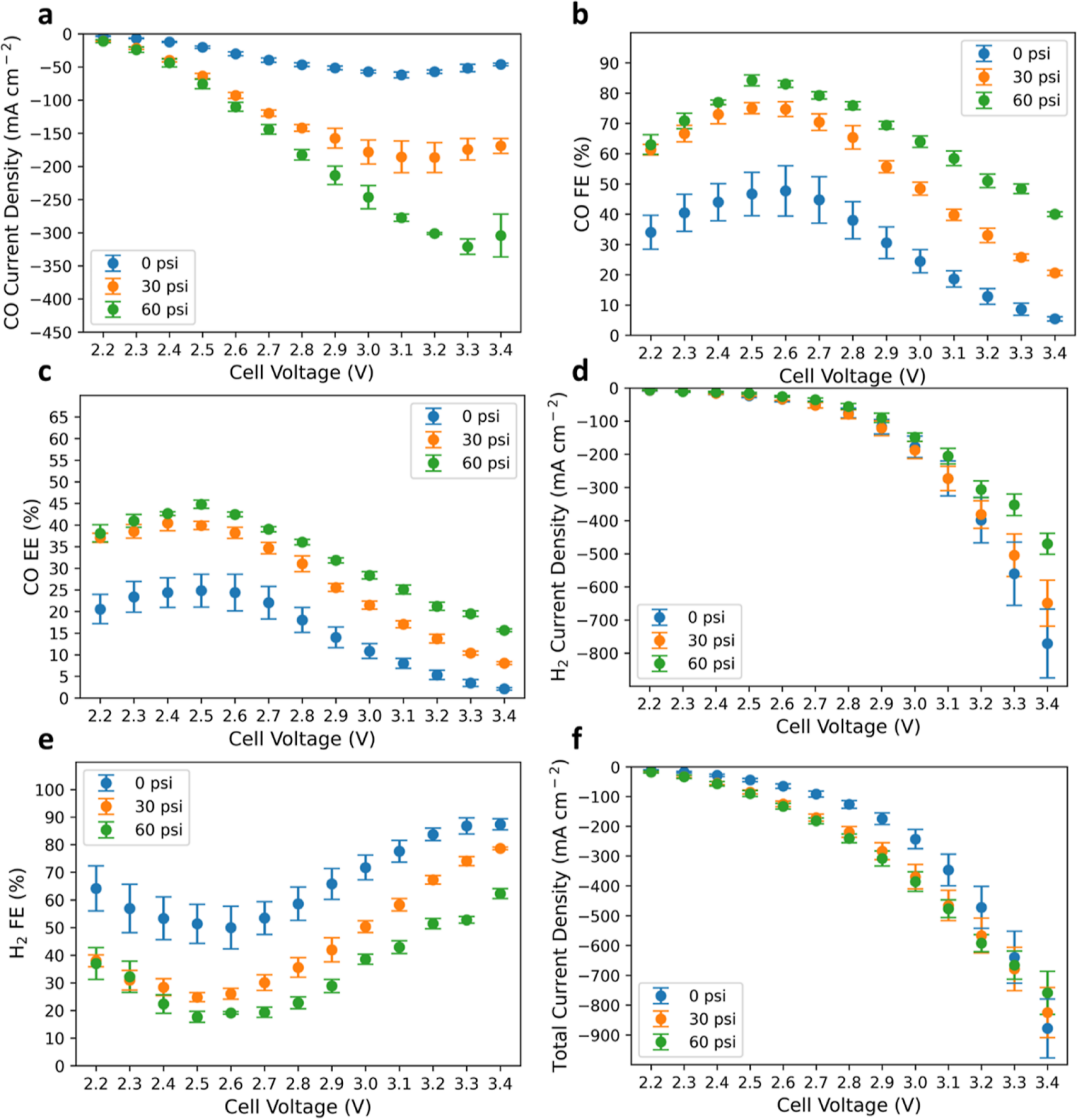
Understanding how e-CO_2_R performance is impacted by various differential pressures applied to the cathode. Dependence of the (a) measured CO partial current density, (b) distribution of CO FE, (c) distribution of CO EE, (d) measured H_2_ partial current density, (e) distribution of H_2_ FE, and (f) total current density as a function of applied voltage. The operating conditions were set to 60 °C at various differential pressures applied only on the cathode, a CO_2_ gas flow rate of 100 sccm, and a DI water flow rate of 100 mL min^–1^. Cathode: 1.3 ± 0.1 mg_Ag_ cm^–2^ and anode: 0.2 ± 0.05 mg_Ir_ cm^–2^. The error bars represent the standard deviation of independent measurements from three identical MEAs.

Operating electrolyzers at elevated pressures to produce pressurized gaseous products can mitigate the need for downstream compression and also reduce the size of piping and system components.^17,39^ Therefore, operating CO_2_ electrolyzers at higher pressure could potentially offer economics benefits. The impact of the cathode differential pressure on e-CO_2_R performance is shown in Figure 3. The cathode differential pressure significantly boosts the CO partial current density, FE, and EE (Figure 3a–c), while the H_2_ partial current density and thus FE are efficiently inhibited (Figure 3d,e) across all the applied voltages. Although the HER thermodynamic equilibrium potential can shift to more negative (*vs* standard hydrogen electrode) at higher pressures, this change has negligible impact on HER for the pressures studied here, as seen for HER in proton-exchange-membrane water electrolyzers (Figure S4). Therefore, this inverse correlation between HER and e-CO_2_R to pressure change could suggest that both reactions can potentially share identical reaction sites on the Ag surface, where if one reaction is dominant, the other reaction can be inhibited. Within a modest voltage range (2.2 to 3.1 V), Figure 3f shows that increasing the pressure from 0 to 30 to 60 lb in^–2^ (psi) shifts the polarization curve toward higher current densities, which is mainly contributed by an increase in e-CO_2_R (Figure 3a vs Figure 3d). At a high applied voltage (>3.2 V) where mass transport of CO_2_ limits e-CO_2_R and leads to CO_2_ deficiency near the catalyst surface, the HER rate increases dramatically and becomes the only option to contribute more currents under a gradual increase in reductive potential if without applying pressure on the cathode. It is also observed that the HFR decreases as the cathode pressure increases (Figure S5). The lower HFR at higher differential pressure is not likely due to BPM deformation, which leads to a possible local membrane thinning effect or contact resistance difference, as the HFRs of the three differential pressures reach identical values at higher applied voltages (3.3 and 3.4 V). Instead, the HFR difference at different differential pressures is driven by the total current (Figure S6), suggested by the higher the total current density, the larger portion of the charges carried by OH^–^ rather than CO_3_^2–^ through AEL, therefore leading to lower HFR.

### Role of Temperature in BPM CO_2_ Electrolyzer

**Figure 4:**
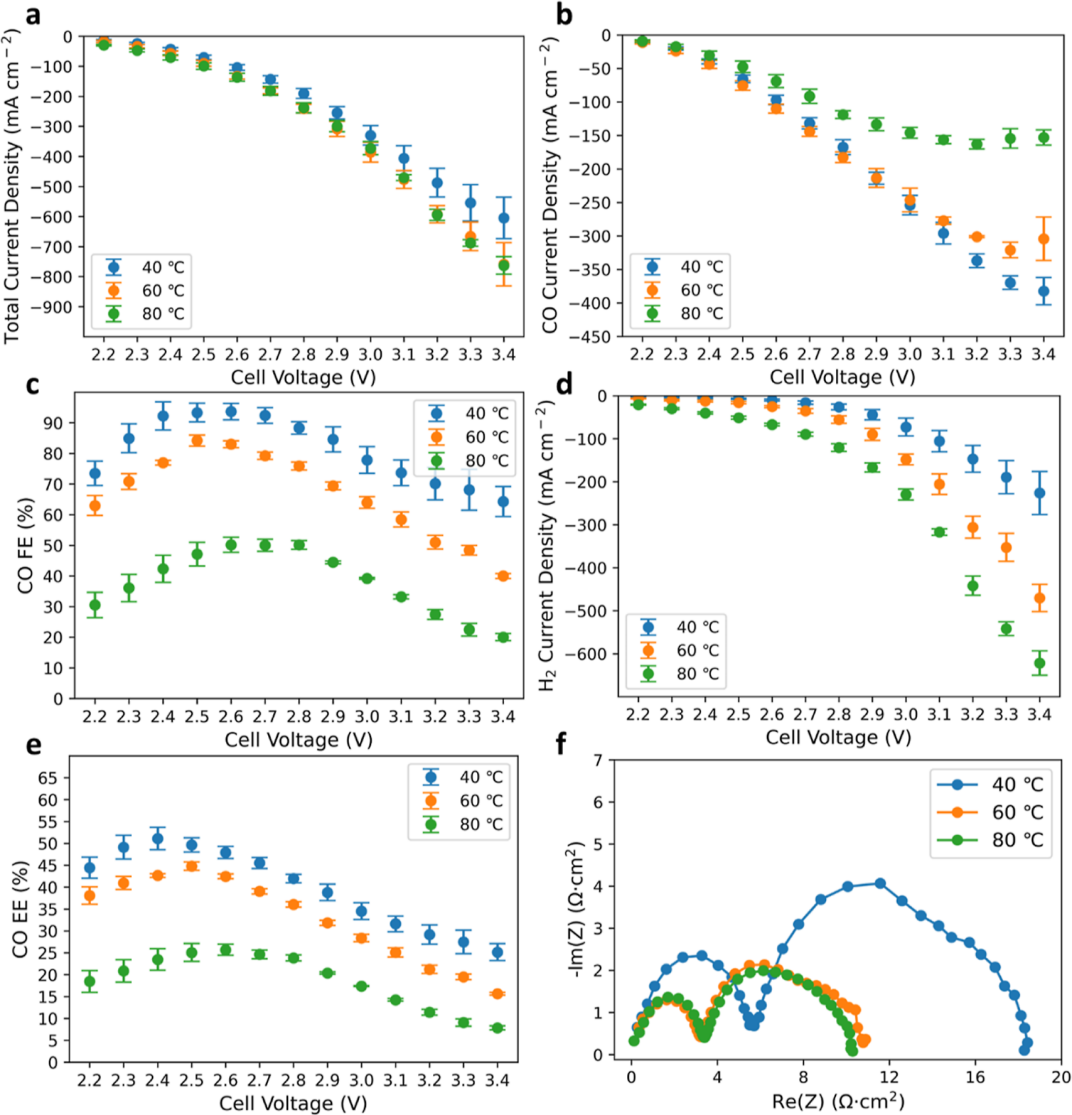
Investigations on how cell temperature impacts the e-CO_2_R performance. Dependence of (a) total current density, (b) measured CO partial current density, (c) distribution of CO FE, (d) measured H_2_ partial current density, and (e) distribution of CO EE as a function of applied voltage. (f) Comparison of the Nyquist plot at three typical temperatures. The operating conditions were set to be three different cell temperatures at 60 psi of differential pressure applied only on the cathode, a CO_2_ gas flow rate of 100 sccm, and a DI water flow rate of 100 mL min^–1^. Cathode: 1.3 ± 0.1 mg_Ag_ cm^–2^ and anode: 0.2 ± 0.05 mg_Ir_ cm^–2^. The error bars represent the standard deviation of independent measurements from three identical MEAs.

While most e-CO_2_R studies have been conducted at room temperature, the benefit of operating polymer–electrolyte MEA devices at elevated temperatures is often expected thanks to enhanced electrode kinetics, electrolyte conductivity, and expedited species transport. This is shown for many electrochemical devices such as fuel cells,^40^ water electrolyzers,^41^ and batteries.^42^ Surprisingly, this benefit is not seen for e-CO_2_R in the BPM electrolyzer, as demonstrated in Figure 4. As the cell temperature increases, the total current increases for a given applied voltage (Figure 4a) due to a significant decrease in the charge-transfer resistance (Figure 4f) and an apparent reduction in HFR (Figure S7). However, the increase in total current density is mostly due to enhanced HER (Figure 4d), while the rate of the CO_2_ to CO reaction (Figure 4b) and efficiencies (Figure 4c,e) are dramatically impaired especially at high operating voltages. Even before e-CO_2_R reaches a mass-transport limitation, the CO partial current density is observed to be lower at higher temperature (80 °C) compared to that at lower temperatures (40 and 60 °C). The negative temperature impact is not likely induced by the anode, as a temperature study in proton-exchange-membrane water electrolyzers (PEMWEs) shows a promotional effect of higher temperature on the oxygen-evolution reaction (Figure S8). The CO partial current density shows a plateauing behavior at a much lower value and applied voltage, suggesting that the transport of CO_2_ to the catalyst surface is impacted at elevated temperatures. Previous studies argue that cathode flooding is a crucial issue for limiting e-CO_2_R current by impacting mass transport due to limited CO_2_ solubility in an aqueous phase.^43,44^ However, this is not expected since higher temperature should increase transport properties and water vapor pressure and thus alleviate flooding.^45^ Unlike previous studies of temperature effects on e-CO_2_R using flow cells with supporting electrolytes, under which the temperature effect can be straightforwardly rationalized by the CO_2_ solubility in the aqueous phase, the difference here is that there is no aqueous supporting electrolyte used.^46,47^ More recent work investigated the temperature impact on e-CO_2_R in a similar MEA device to this study; however, the detailed temperature impact on adsorption was not well understood.^30^ We hypothesize that the temperature impacts the CO_2_ adsorption on the catalyst surface at elevated temperatures as e-CO_2_R is likely to occur among the heterogeneous interfaces of catalyst, polymer–electrolyte, and absorbed CO_2_ gas.

**Figure 5:**
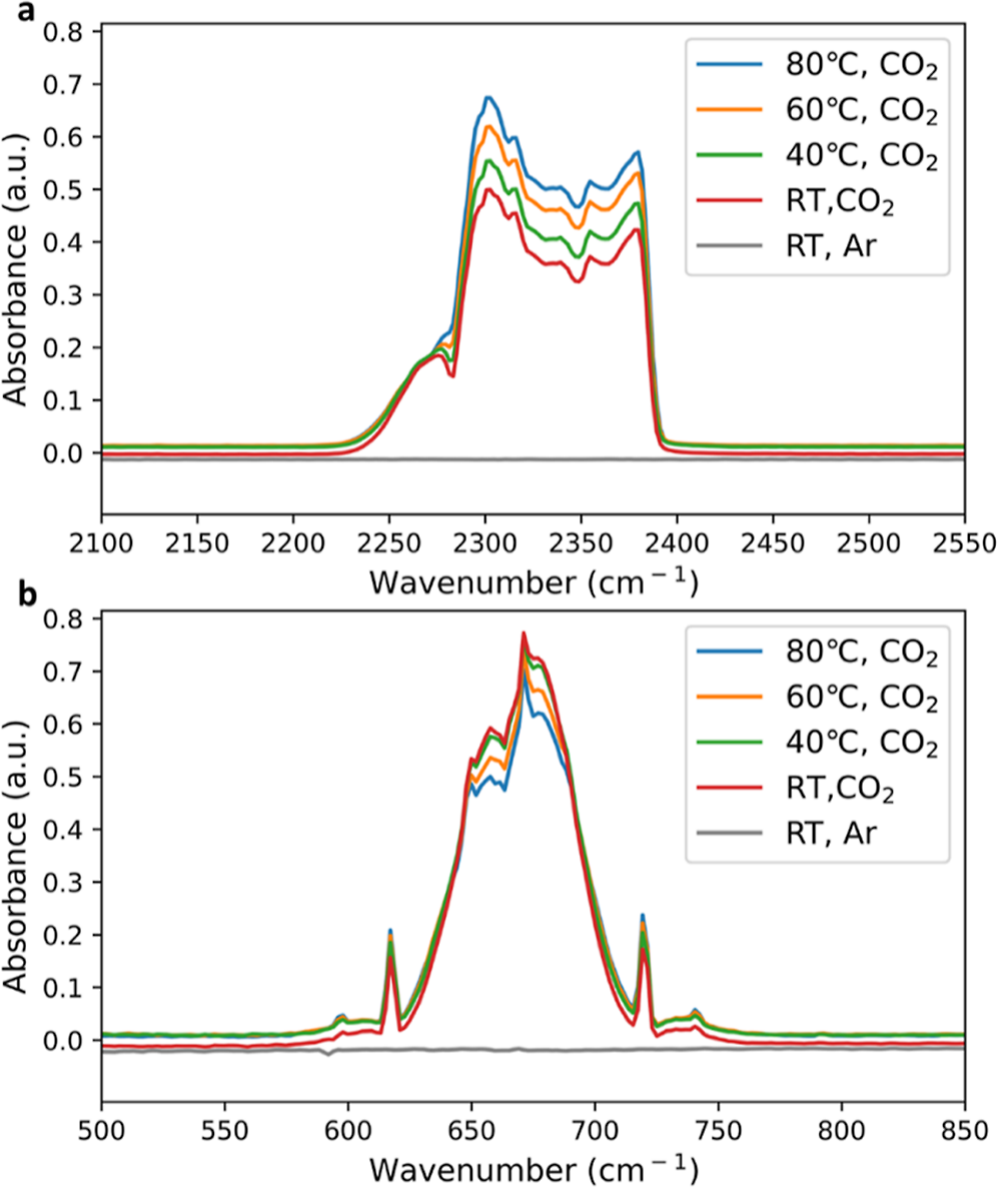
Investigations on CO_2_absorption on the Ag surface. (a) Temperature-dependent CO_2_ asymmetric stretching on the Ag surface and (b) temperature-dependent CO_2_ symmetric bending on the Ag surface.

As the CO_2_ to CO reaction involves the C=O bond cleavage,^48^ the scissoring vibration of CO_2_ molecule is likely to promote the reaction. To verify this hypothesis, diffuse-reflectance infrared Fourier transform spectroscopy (DRIFTS) was used to probe the CO_2_ adsorption behavior on the Ag catalyst surface at various temperatures. The result shows that even though the CO_2_ asymmetrical stretching is enhanced at higher temperatures (Figure 5a), the symmetrical bending (scissoring) mode on the Ag surface is inhibited as the increase of temperature (Figure 5b), therefore, potentially impacting CO_2_ reduction to CO. These results suggest that a higher temperature preferentially favors HER compared to e-CO_2_R by altering CO_2_ adsorption behavior on the catalyst surface. To leverage the kinetic benefits of higher operating temperatures, as shown by lower charge-transfer resistance for both the anode and cathode (Figure 4f), one can expect that high cathode differential pressure is needed to help with CO_2_ transport and adsorption on the catalyst surface.

### CO_2_ Crossover and Durability of the Water-Fed BPM Electrolyzer

The CO_2_ crossover rate was measured by quantifying the level of CO_2_ at the anode exhaust. The total current density increases the CO_2_ crossover rate, indicating the increase of the CO_2_ concentration at the bipolar junction, which promotes diffusional crossover to the anode side (Figure S9). However, this diffusional CO_2_ crossover rate is significantly lower compared to that in a conventional alkaline-based CO_2_ electrolyzer. We compared crossover-CO_2_ with the amount of CO_2_ converted to CO (converted-CO_2_). The ratios between crossover-CO_2_ and converted-CO_2_ would be 2 and 1 for HCO_3_^–^ and CO_3_^2–^ as charge carriers in conventional alkaline-based devices, respectively. The measured ratio herein is 2 orders of magnitude lower, even with the cathode differential pressure, suggesting effective suppression of CO_2_ crossover (Figure S10). It is also worth noting that the ratio decreases as the total current density increases (Figure S10), which also indicates the change of charge carrier from HCO_3_^–^/CO_3_^2–^ to OH^–^ thanks to the feed of pure water to minimize the aqueous phase OH^–^, therefore preventing the fast formation of HCO_3_^–^/CO_3_^2–^.

**Figure 6:**
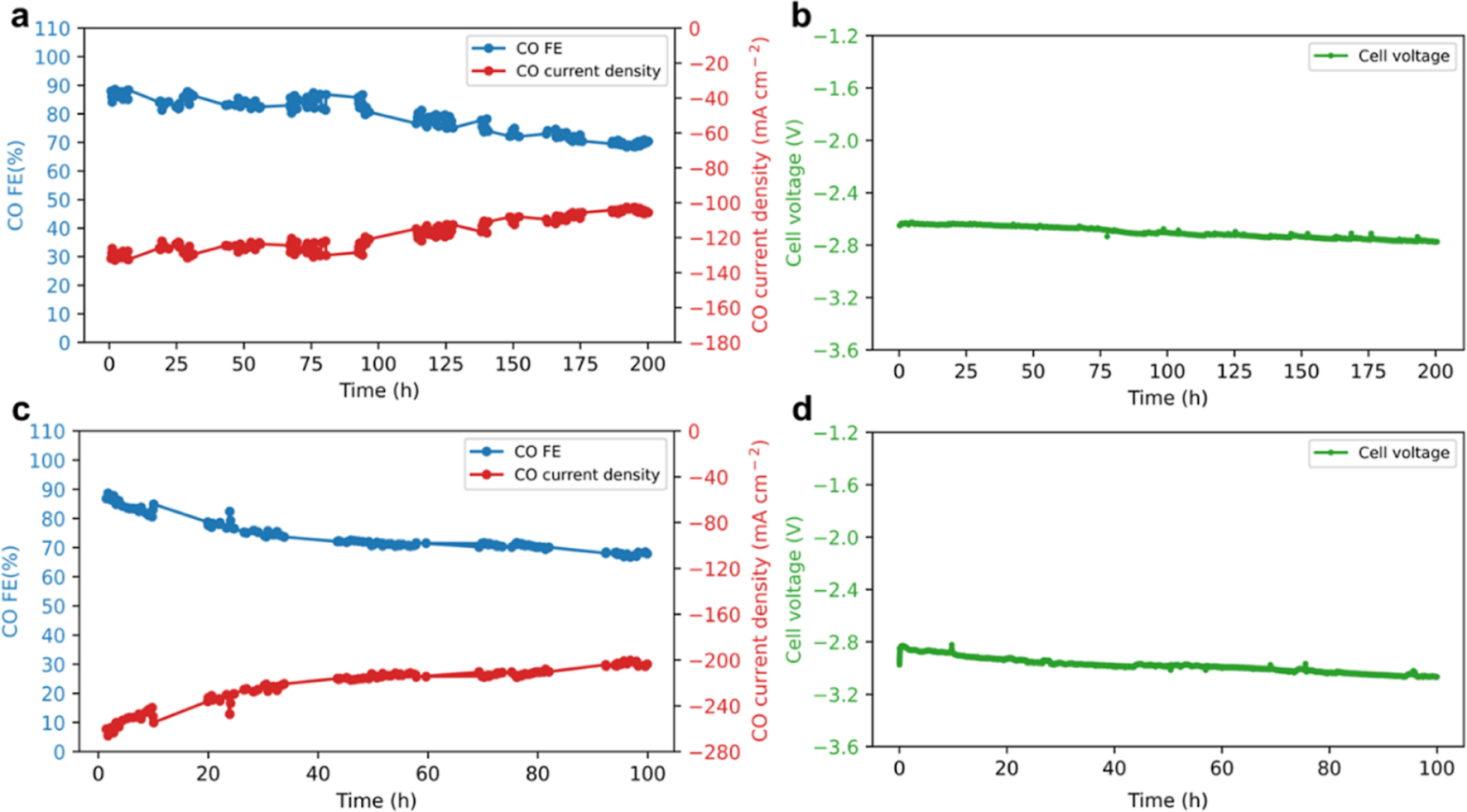
Investigations on the durability of the BPM CO_2_ electrolyzer. Short-term durability of the BPM CO_2_ electrolyzer: (a,b) CO FE, current density, and cell voltage at a total current of 150 mA cm^–2^ and (c,d) CO FE, current density, and cell voltage at a total current of 300 mA cm^–2^. The operating conditions were set to be 40 °C cell temperatures at 60 psi of differential pressure applied only on the cathode, a CO_2_ gas flow rate of 100 sccm, and a DI water flow rate of 100 mL min^–1^. Cathode: 1.3 ± 0.1 mg_Ag_ cm^–2^ and anode: 0.2 ± 0.05 mg_Ir_ cm^–2^. Small voltage fluctuation is due to replenishing water to the heating bath for the anode.

As the BPM CO_2_ electrolyzer performed the best at 40 °C, this condition was chosen for longer-term durability studies. Two continuous durability tests were conducted at total current densities of 150 mA cm^–2^ and 300 mA cm^–2^ for 200 and 100 h, respectively. The water-fed BPM CO_2_ electrolyzer starts at a cell voltage of 2.65 V and a CO FE of 87% and shows an average voltage decay rate of 0.61 mV h^–1^ and a CO partial current density decay rate of 0.13 mA_CO_ cm^–2^ h^–1^ for the 200 h durability test (Figure 6a,b), while it shows an average voltage decay rate of 2.1 mV h^–1^ and a CO partial current density decay rate of 0.57 mA_CO_ cm^–2^ h^–1^ for the 100 h durability test (Figure 6c,d). After the durability test, the cross-sectional SEM images of the BPM show no visible delamination between AEL and PEL (Figure S11), indicating the superiority of asymmetrical design in suppressing BPM interfacial contact loss during operation. This is further supported by negligible changes in HFR for the BPM CO_2_ electrolyzer at the beginning-of-life (BOL) and end-of-life (EOL) after durability tests (Figure S12). Instead, the charge-transfer resistance shows nearly 50% enhancement at EOL compared to that at BOL by Nyquist plots, which mostly explains the overall performance decay. The root cause of the electrode deactivation is possibly driven by catalyst structural changes and ionomer degradation under cathodic operation, both of which can deteriorate the catalyst/polymer–electrolyte interfaces. The SEM images of cathode catalyst layers show significant catalyst agglomeration at EOL compared to that at BOL (Figure S13), which potentially results from catalyst dissolution and redeposition under cathodic potential.^49^ In addition, X-ray photoelectron spectroscopy (XPS) of the EOL cathode catalyst layer shows severe loss of nitrogen from the cationic functional headgroup (Figure S14). AEIs are more prone to oxidative decay under anodic potential;^50^ however, degradation could also occur if exceeding the electrochemical stability window even under reductive potential. The performance metrics of this work is compared to that of previously reported BPM CO_2_ electrolyzers for CO production (Table S1),^29,31,51^ which indicates enhanced efficiency and durability, although differences in the testing condition and the material should be noted. Pure-water-fed BPM systems are likely to underperform compared to that of electrolyzers fed with supporting electrolytes^52^ due to the promotional effects of cations and enhanced electrochemical active surface area by forming nearly ubiquitous catalyst/aqueous–electrolyte interfaces; however, the benefit in avoiding precipitation and CO_2_ crossover could overweight initial performance. These results demonstrate the potential commercial applicability of the CO_2_ electrolyzer using this design, although future research is needed to further improve performance and durability especially at higher operating currents.

## Conclusions

In summary, this work proposes a pure-water-fed forward-bias BPM CO_2_ electrolyzer to address the challenges of CO_2_ crossover and salt precipitation. By manipulating operational conditions, including temperature and pressure, this work demonstrates CO FE over 80% with a CO partial current density over 200 mA cm^–2^ at less than 3.0 V and 100 h continuous operation at a total current density of 300 mA cm^–2^. The proton crossover and CO_2_ adsorption behavior are believed to be the two critical factors that impact the product distribution and efficiencies. Overall, this pure-water-fed asymmetrical BPM electrolysis strategy could pave the way for the possible commercial deployment of CO_2_ electrolyzers.

## Experimental Section

### Materials

Silver nanopowder (APS 20–40 nm, 99.9% metal basis) was purchased from Thermo Fisher Scientific Inc. Iridium oxide (IrOx) on carbon (ELC-0110) was purchased from TANAKA (TKK). PiperION A ionomer dispersion (5 wt % in ethanol) was purchased in a bicarbonate form from Versogen, Inc. Nafion dispersion (D521, 5 wt % in ethanol) was purchased from Ion Power. Polytetrafluoroethylene (PTFE) dispersion (60 wt % in water) was purchased from Sigma-Aldrich. Ethanol (ACS reagent, 99.5%) and n-propanol (nPA, ACS reagent 99.5%) were purchased from Sigma-Aldrich. DI water (18.2 MΩ·cm) was produced in-house using a Milli-Q (EMD Millipore). Nafion 117 membranes were purchased from Ion Power. Carbon paper gas-diffusion layers (Freudenberg H23C8) were purchased from the Fuel Cell Store. Platinized sintered-titanium porous-transport layers were purchased from Mott Corporation. The PTFE gasketing material was purchased from CS Hyde.

### Ink Preparation and Electrode Fabrication

The anode catalyst ink was prepared by tip sonicating (Ultrasonic Processor, Cole Parmer) a dispersion of 50 mg of supported IrOx, 116 mg of Nafion dispersion, 10 g of DI water, 16.08 g of nPa, and 7.89 g of ethanol for 35 min in an ice bath. The amplitude for sonication was set to 38%. For the cathode catalyst ink, 300 mg of Ag nanopowder, 2 g of DI water, and 14.4 g of nPa were first tip sonicated with an amplitude of 38% for 15 min in an ice bath. After adding 600 mg of PiperION A ionomer dispersion [0.1 (g/g) ionomer/catalyst] and 60 mg of PTFE dispersion (accounting for 10 wt % PTFE in a catalyst layer), the ink was sonicated in an ice bath in an ultrasonic cleaner (CPX2800H, Branson) for 45 min. The anode catalyst ink was spray coated onto a Nafion 117 membrane by using an automated coating machine (ExactaCoat, Sono-Tek Corp.). The membrane was placed on a heated vacuum plate, which was set at a temperature of 50 °C. A loading of 0.2 ± 0.05 mg_Ir_ cm^–2^ was targeted by spraying a volume of approximately 10 mL of ink at a pump rate of 0.25 mL min^–1^ on a geometrical area of 10 cm^2^. The anode catalyst loading was determined by X-ray fluorescence spectroscopy (Bruker). The exact loadings were calculated based on a calibration curve measured from six Ir standard loadings purchased (Micromatter Technologies Inc.) along with a blank standard (0 mg cm^–2^). The cathode catalyst ink was sprayed manually onto a carbon paper gas diffusion layer using an airbrush (Eclipse, ANEST Iwata-Medea). The carbon-paper gas-diffusion layer was stamped to a geometrical size of 30.25 cm^2^. During spraying, the ink was frequently dried by placing the electrode on a heating plate set to 75 °C. The loading of the silver catalyst was determined from the difference in weight of the carbon paper gas diffusion layer before and after spraying and was calculated to be 1.3 ± 0.1 mg_Ag_ cm^–2^.

### Fabrication of Asymmetric BPMs

To have a flat surface for spraying the asymmetric BPMs, the catalyst-coated membranes (CCMs) were soaked in DI water for 15 min and then put between two metal plates, of which one plate had a window of 10 cm^2^. The CCM was then gently dried with a N_2_ gas flow before addition of the AEL. For the preparation of the AEL, the ionomer dispersion was first mixed with ethanol. The diluted solution was then manually spray coated onto a 10 cm^2^ geometrical area of the uncoated CCM half side. The spray solution was frequently dried by using the N_2_ gas stream from the airbrush. To obtain different thicknesses of the AEL, the dilution ratio was adopted. Here, 0.25, 0.5, or 0.75 g of ionomer dispersion was mixed with 3 g of ethanol. The thickness of the AEL was determined from cross-sectional SEM images of the asymmetric BPM. To account for the reproducibility limitations of manual airbrushing, the thickness is indicated with an uncertainty of 20% of the measured thickness.

### Cell Assembly and Testing

A commercial electrochemical test cell (Fuel Cell Technology) was used to perform electrolysis experiments. The GDEs were cut into 1 cm^2^ squares using a die and put into a 0.5 M CsHCO_3_ solution for 1 h followed by intensive rinse in DI water and were then gently dried using lint-free wipes before assembling the electrochemical cell. Maintaining aqueous electrolyte free for the cathode GDE is critical as residual cations can impact the protonic conductivity of the Nafion membrane. The catalyst-coated BPMs were soaked in DI water for at least 1 h. The porous-transport layers were also cut into 1 cm^2^ squares by laser cutting and washed with DI water before placing them between the anodic flow field and the IrOx-coated membrane side. A 10 mL gasket was used as a spacer between the CCM and the anodic flow field. On the cathodic half side, 2 and 5 mL gaskets were used as spacers to maintain a compression ratio of the GDE of approximately 25%. After the layers were assembled, the cell was compressed by tightening the screws with a torque wrench set at 40 ft-lb. Cell testing was carried out using a biologic VSP potentiostat with a 20 A booster (VMP3B-20). Polarization curves were assessed in potentiostatic operation by keeping each voltage constant for 15 min. Electrochemical impedance spectroscopy (EIS) was performed after every tested voltage with a sinus amplitude of 10 mV in a frequency range from 100 mHz to 1 MHz and six frequencies per decade. Using the EIS data, HFR was determined from the Nyquist plot by reading out the first point of the semicircle. For all experiments, CO_2_ with a purity of 99.995% was fed at a flow rate of 100 sccm. The temperature of the test cell was regulated by using electrical resistance heating. The gas pressure was controlled with a backpressure regulator. A gas chromatograph (8610C, SRI Instruments) was used to quantify the product gas composition. Two injections were performed after 5 and 11 min of the electrolysis experiment at each cell voltage, respectively. All reported values are an average of the data assessed at the two-time injections. The flow rate of the gas stream coming out of the electrolyzer was measured by using a digital mass-flow meter (MFM, Alicat). Each experimental condition was measured three times independently.

To investigate the crossover of gases from the cathode to the anode, the samples of the gas outlet of the anode DI water bottle were injected into the gas chromatograph using a nitrogen flow (∼100 sccm) to flush gas from the head space of the bottle. The nitrogen flow rate was also monitored by using a digital MFM (Alicat). The CO_2_ crossover rate was calculated based on the gas chromatograph CO_2_ signal and the nitrogen flow rate. The total duration of the crossover measurement lasts for a total of 15 min/voltage × 13 voltages = 195 min continuously.

### Product Quantification and Data Analysis

FE FE_*i*_ for the reduction products *i* = CO, H_2_, and CH_4_ was calculated from the current *I*, the charge number z_*i*_, the product flow rate *V̇*_P,S_ at standard conditions, the molar concentration *x*_*i*_, the ideal gas constant *R*, the Faraday constant *F*, the pressure *p*_S_, and the temperature *T*_S_.
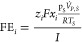
1

EE for the main CO_2_ reduction product CO EE is calculated from
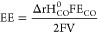
2where Δ*rH*_CO_^0^ is the enthalpy change of Reaction 1 (Δr*H*_CO_^0^ = 283 kJ mol^–1^)^53^ and *V* is the cell potential.

The ratio between crossover-CO_2_ and converted-CO_2_ is defined as
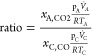
3where A and C denote the anode and cathode sides, respectively.

### X-ray Photoelectron Spectroscopy

X-ray photoelectron spectra were collected by using an XPS Kratos Axis Ultra DLD system with a monochromatic Al Kα source (*h*ν = 1486.6 eV). Spectral analysis was performed using CasaXPS software, and binding energies were calibrated to the C 1s signal at 284.8 eV.

### Scanning Electron Microscopy

SEM images were collected using a JEOL JSM 7500F. To image the cross-section of the membrane layers, the samples were fractured after submerging in liquid nitrogen.

### Diffuse-Reflectance Infrared Fourier Transform Spectroscopy

DRIFTS was performed using a Thermo Nicolet 6700 spectrometer with a mercury–cadmium–telluride detector and a KBr window-equipped cell. The sample was pretreated at 100 °C for 1 h under a 30 sccm Ar flow. Once the sample cooled to room temperature, CO_2_ was introduced at a flow rate of 30 sccm. Spectra were recorded while ramping the temperatures to 40, 60, and 80 °C after confirming CO_2_ saturation on the sample surface.

## Supporting Information Available

Experimental details, electrochemical measurements and material characterizations, performance, and durability comparison among different bipolar CO_2_ electrolyzers, and SEM images (PDF)
