# Supplementary material for: Pure-Water-Fed Forward-Bias Bipolar Membrane CO2 Electrolyzer
Source: ACS Appl Mater Interfaces. 2024 May 7;16(19):24649–59. doi: 10.1021/acsami.4c02799 (PMC11103649; doi:10.1021/acsami.4c02799)
Supplement: Supplementary file 1 — am4c02799_si_001.pdf [file am4c02799_si_001.pdf]

Supporting Information

Pure-water-fed forward-bias bipolar membrane CO_2 electrolyzer

Matthias Heßelmann[1,2], Jason Keonhag Lee[1], Sudong Chae[1], Andrew Tricker[1], Robert Gregor Keller[2], Matthias Wessling[2,3], Ji Su[1], Douglas Kushner[1], Adam Z Weber[1] and Xiong Peng[1,*]

[1] Energy Technologies Area, Lawrence Berkeley National Laboratory, Berkeley, CA, 94720, USA

[2] RWTH Aachen University, Chemical Process Engineering, Forckenbeckstr. 51, 52074 Aachen, Germany.

[3] DWI Leibniz-Institute for Interactive Materials, Forckenbeckstr. 50, 52074 Aachen, Germany

*Corresponding author

E-mail: xiongp@lbl.gov (Xiong Peng)

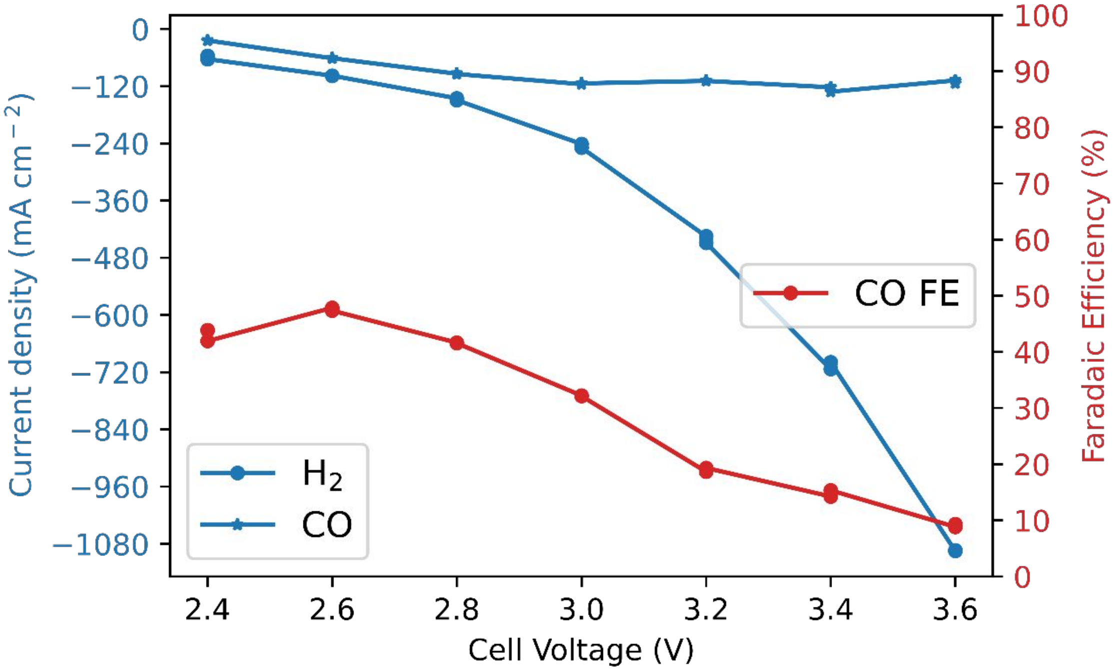

Figure S1. The e-CO_2RR performance of using anion exchange ionomer (AEI) coated gas diffusion electrode (GDE) coupled with a Nafion 117 membrane and iridium anode. The blue lines are the CO partial current density (star) and H_2 partial current density (circle). The red line is the CO Faradaic efficiency (FE). There are two data points plotted for each applied voltage. The operating conditions were set to a temperature of 60°C, a differential pressure of 60 psi applied only on cathode, a CO_2 gas flow rate of 100 sccm, and a DI water flow rate of 100 ml min^{-1}.

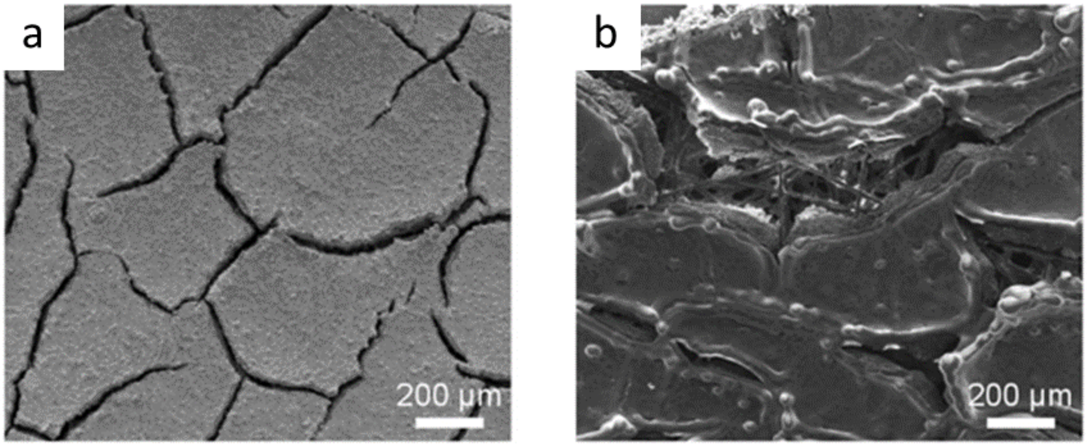

Figure S2. Scanning electron microscopy images of a) the catalyst layer surface without AEI coating and b) the AEI-coated catalyst layer surface for gas diffusion electrodes.

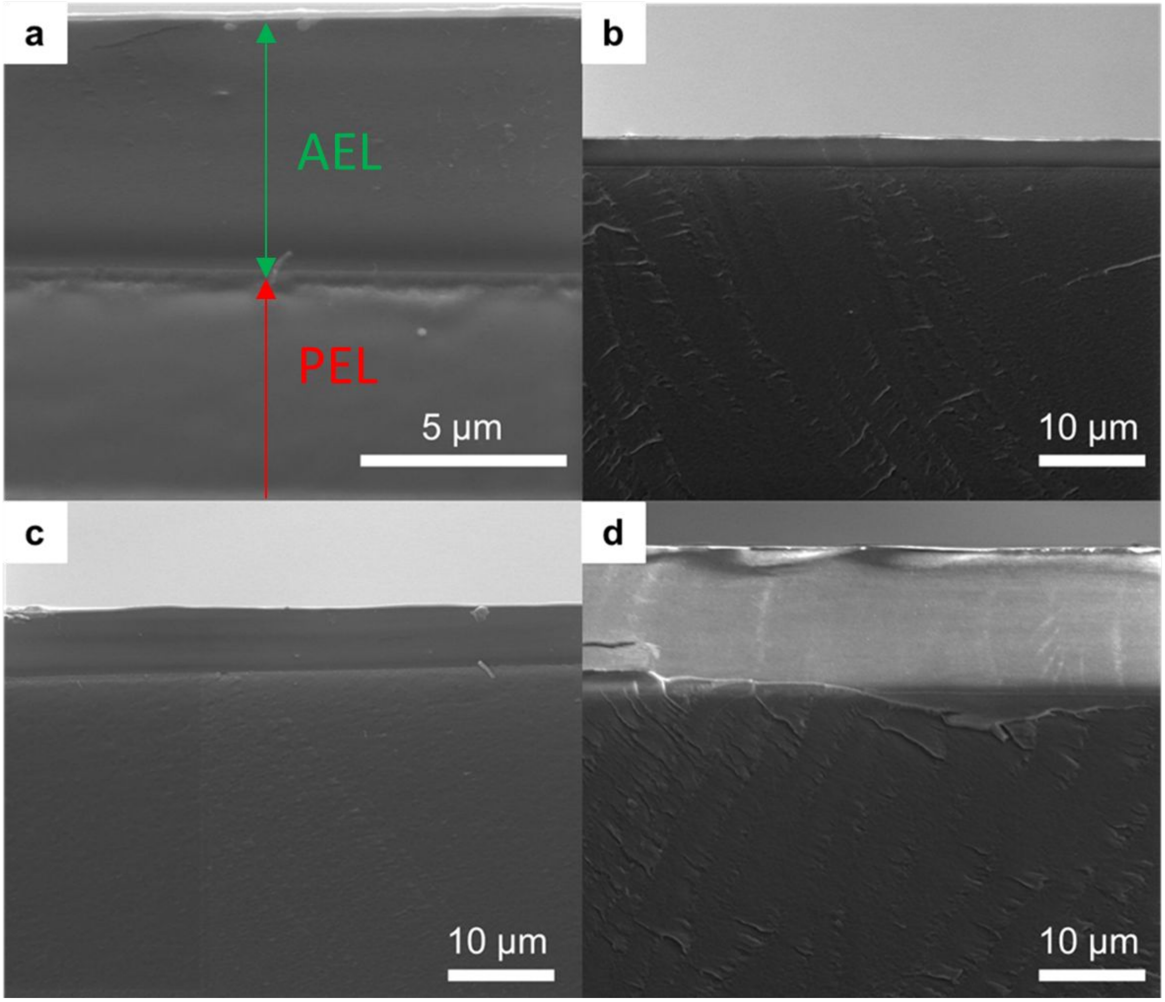

Figure S3. Scanning electron microscopy images of the asymmetrical BPM. a) the intimate bipolar junction formed at the AEL and PEL interface. Cross section SEM images of the BPM at various AEL thickness: b) 3±0.6 µm, c) 6±1.2 µm and d) 14±2.8 µm.

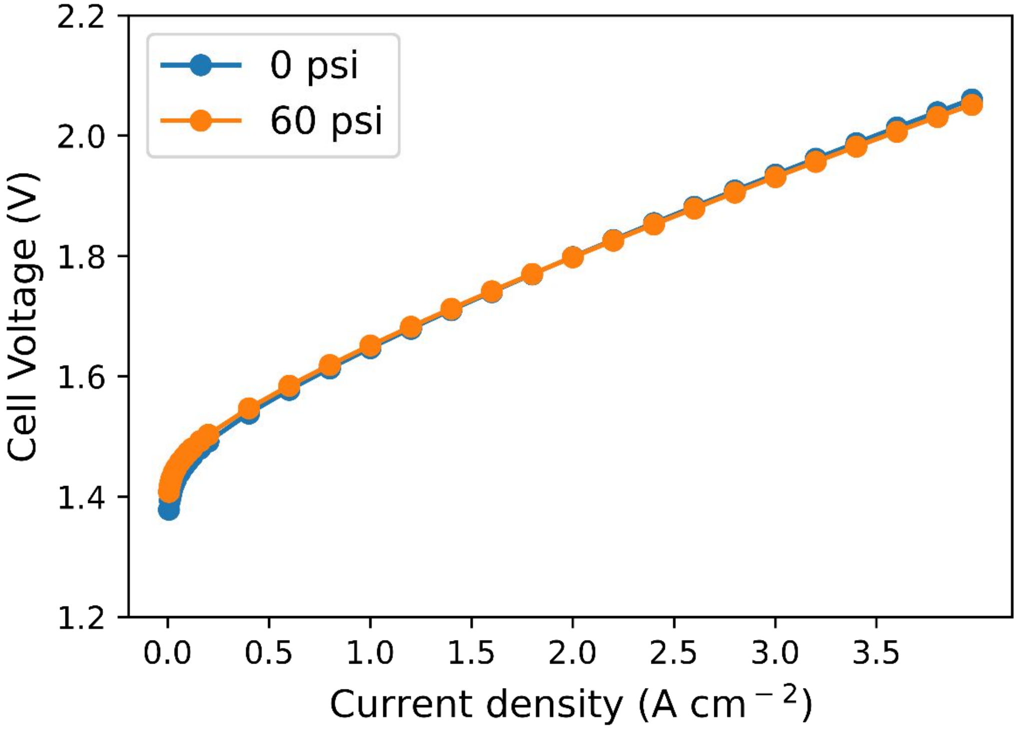

Figure S4. Impact of cathode differential pressure on hydrogen evolution reaction in a proton exchange membrane water electrolyzer. As the cathode differential pressure (gauge pressure: 0 psi → 60psi) increases, the electrolyzer performance stays relatively unchanged, which indicates that the cathode pressure has a negligible impact on the hydrogen evolution reaction for the cathode. Cathode: 0.1 mgPt/cm2, anode: 0.2 ± 0.05 mgIr/cm2, Nafion 117 membrane with deionized water fed to the anode only. Cell temperature: 80 ℃.

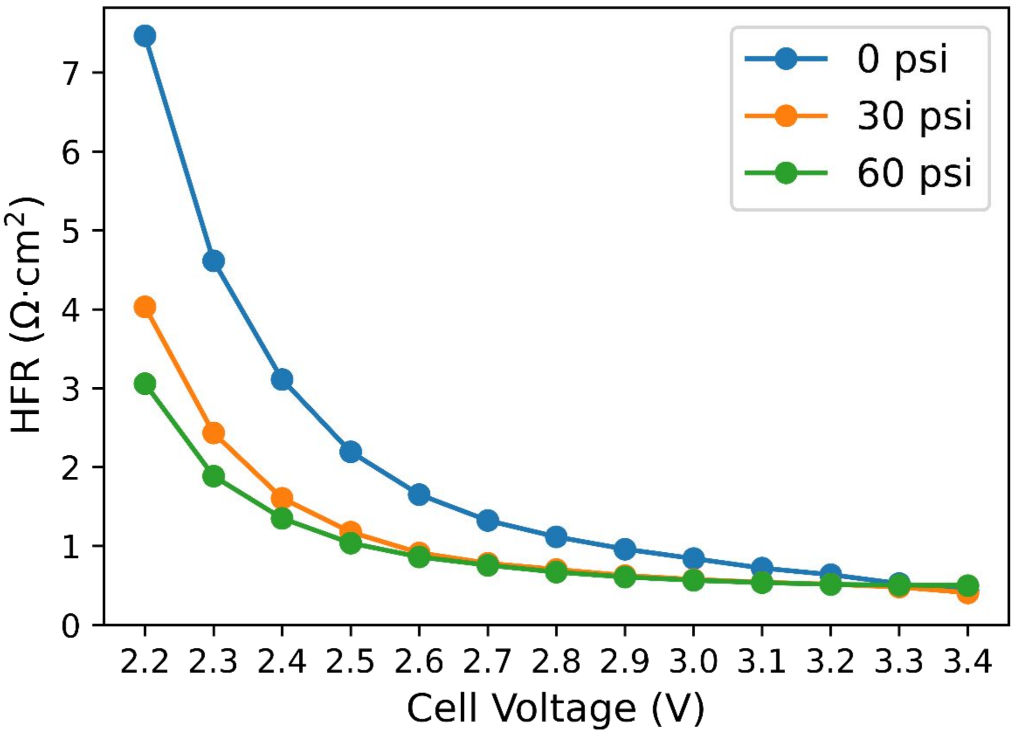

Figure S5. Comparison of high frequency resistance measured at various cathode differential pressure across all applied voltages in a water-fed BPM CO_2 electrolyzer. The operating conditions were set to 60°C at various differential pressures applied only on the cathode, CO_2 gas flow rate of 100 sccm, and DI water flow rate of 100 ml min $^{-1}$. Cathode: 1.3 ± 0.1 mg_{Ag}/cm^2 and anode: 0.2 ± 0.05 mg_{Ir}/cm^2.

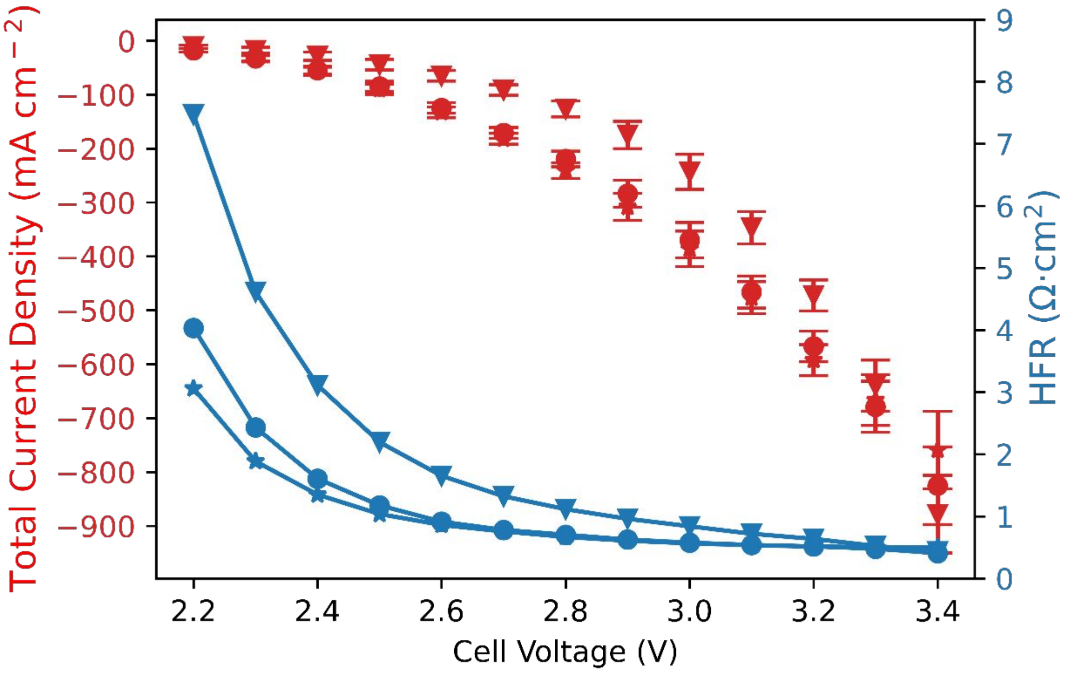

Figure S6. Correlation between high-frequency resistance and total current densities measured at various cathode differential pressures (triangle: 0 psi, circle: 30 psi and star: 60 psi) across all applied voltages in a water-fed BPM CO_2 electrolyzer. The operating conditions were set to 60°C at various differential pressures applied only on the cathode, CO_2 gas flow rate of 100 sccm, and DI water flow rate of 100 ml min^{-1}. Cathode: 1.3 ± 0.1 mg_{Ag}/cm^2 and anode: 0.2 ± 0.05 mg_{Ir}/cm^2.

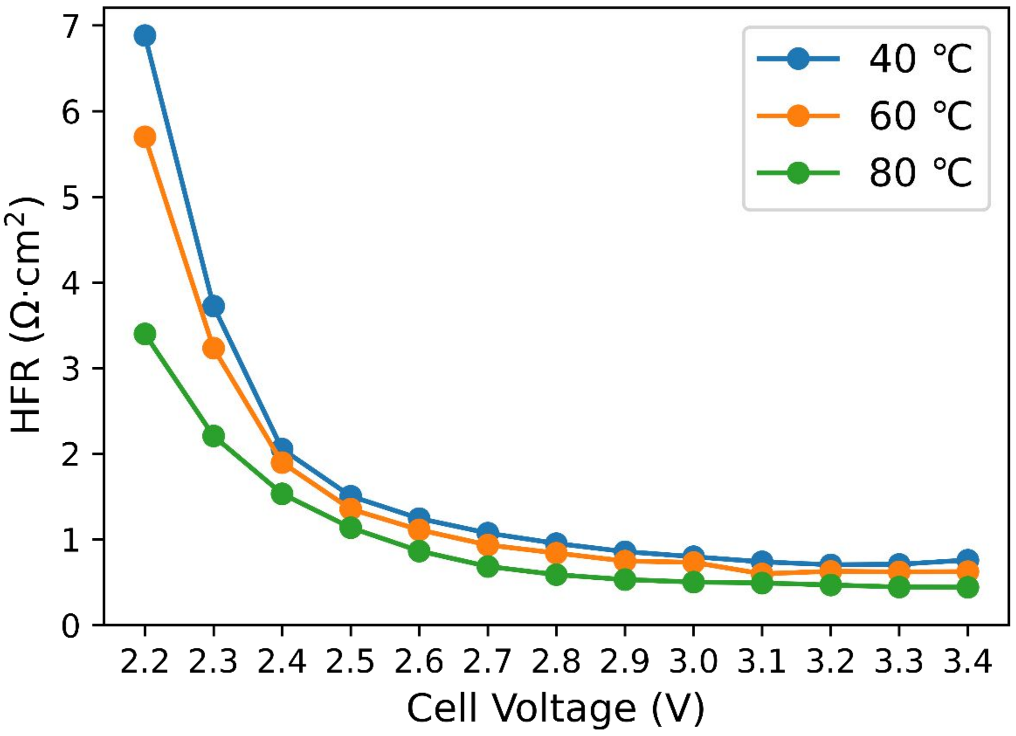

Figure S7. Comparison of high frequency resistance measured at various cell temperatures across all applied voltages in a water-fed BPM CO_2 electrolyzer. The operating conditions were set to be three different cell temperatures at 60 psi of differential pressure applied only on cathode, CO_2 gas flow rate of 100 sccm, and DI water flow rate of 100 ml min $^{-1}$. Cathode: 1.3 ± 0.1 mg_{Ag}/cm^2 and anode: 0.2 ± 0.05 mg_{Ir}/cm^2.

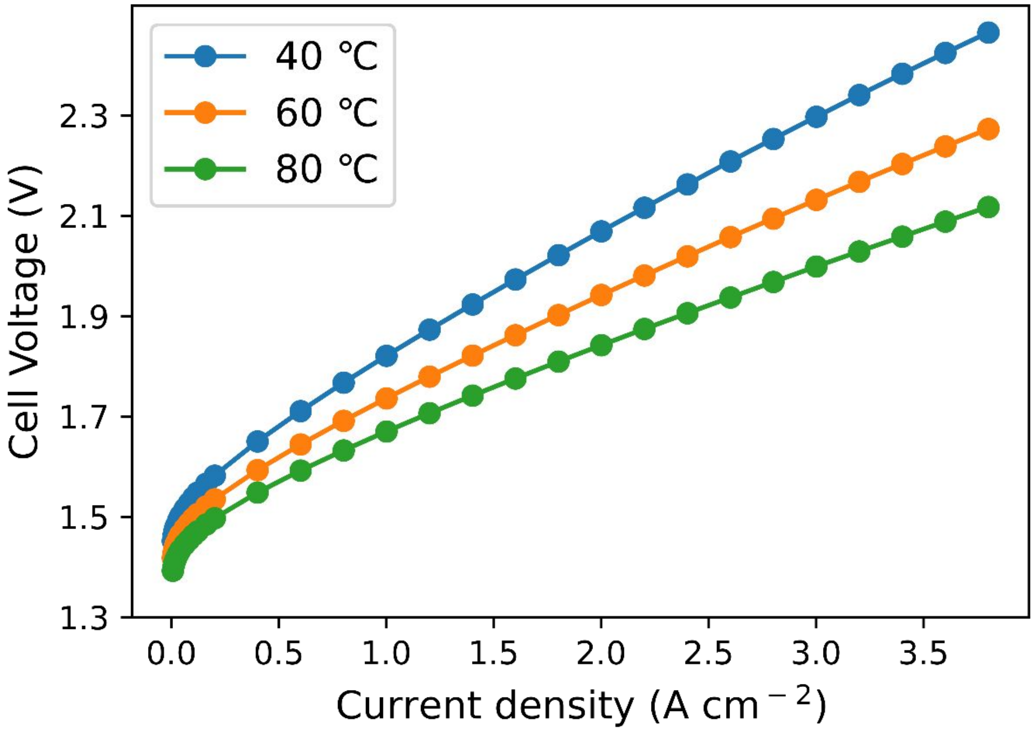

Figure S8. Impact of operating temperature on oxygen evolution reaction for proton exchange membrane water electrolysis. As the cell temperature increases, the electrolyzer performance increases, which indicates the promotional effect of temperature on the anode. Cathode: 0.1 mgPt/cm2 at ambient pressure, anode: 0.4 mgIr/cm2, Nafion 117 membrane with deionized water fed to the anode only.

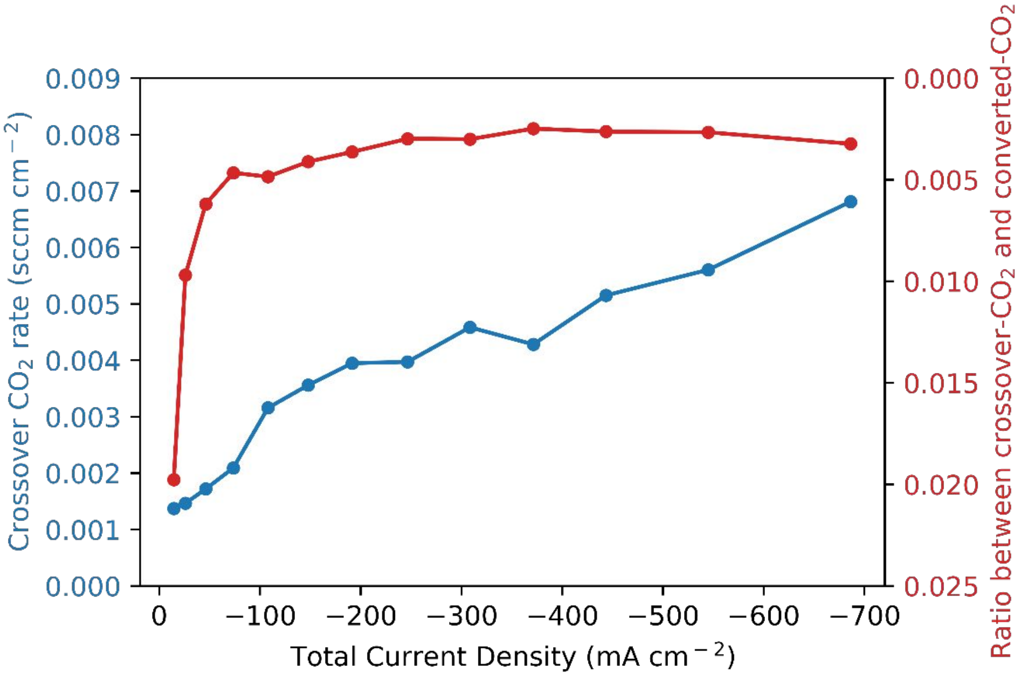

Figure S9. The total CO_2 crossover rate (sccm cm^{-2}) in a water-fed BPM CO_2 electrolyzer as a function of total current density and molar ratio between the crossover CO_2 and the CO_2 converted into CO. The operating conditions were set to be a cell temperature of 40 ℃ at 60 psi of differential pressure applied only on the cathode, CO_2 gas flow rate of 100 sccm, and DI water flow rate of 100 ml min^{-1}. Cathode: 1.3 ± 0.1 mg_{Ag}/cm^2 and anode: 0.2 ± 0.05 mg_{Ir}/cm^2.

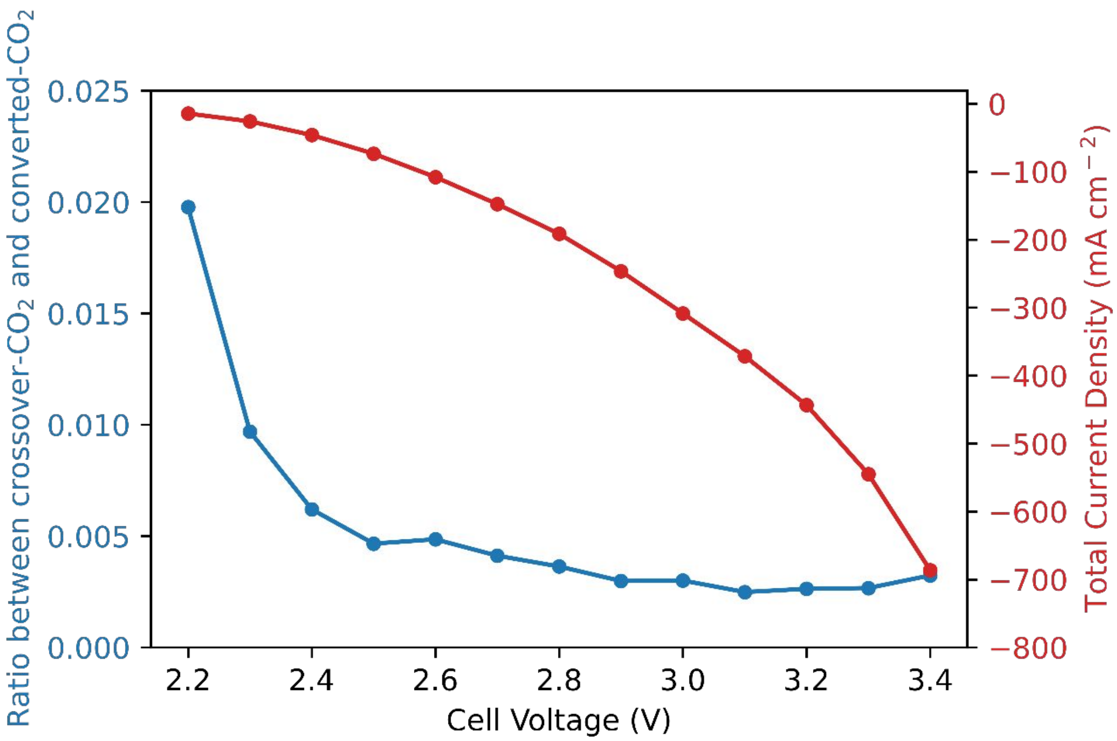

Figure S10. The molar ratio between the crossover CO_2 and the CO_2 converted into CO in a water-fed BPM CO_2 electrolyzer at different applied cell voltage and total current densities. The operating conditions were set to be a cell temperature of 40 °C at 60 psi of differential pressure applied only on the cathode, CO_2 gas flow rate of 100 sccm, and DI water flow rate of 100 ml min $^{-1}$. Cathode: 1.3 ± 0.1 mg_{Ag}/cm^2 and anode: 0.2 ± 0.05 mg_{Ir}/cm^2.

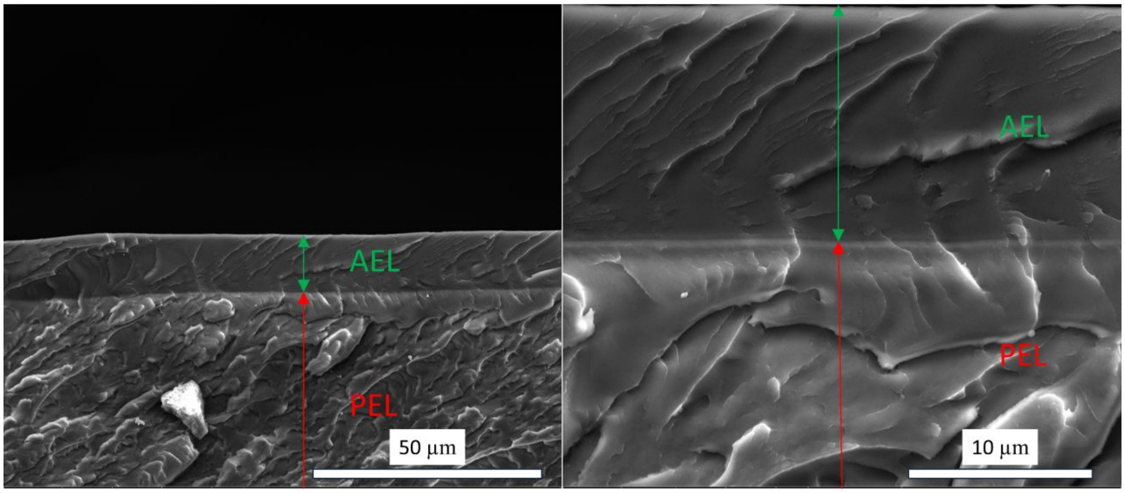

Figure S11. Scanning electron microscopy images of the asymmetrical BMP interface between AEL and PEL after 200-h of durability test at a total current density of 150 mA cm^{-2}.

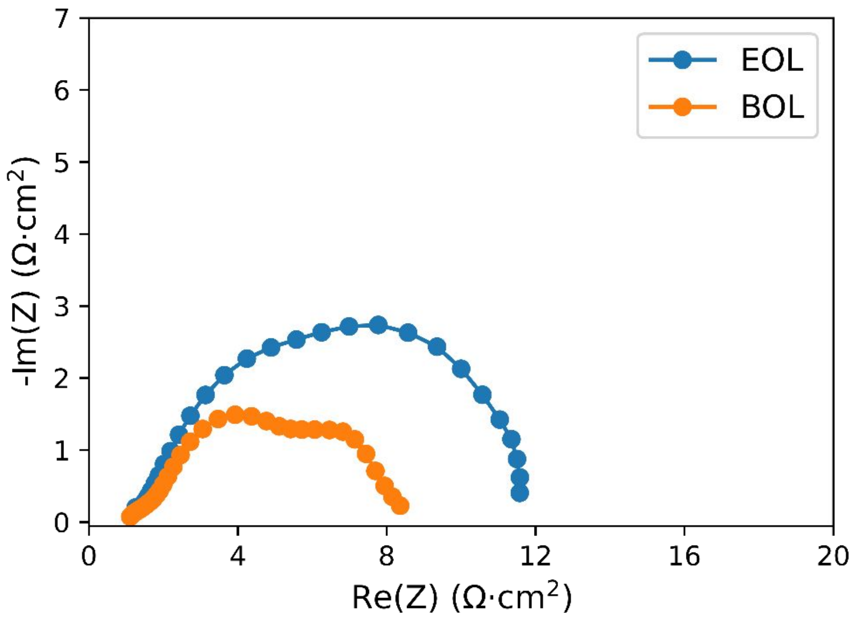

Figure S12. Nyquist of the water-fed BPM CO_2 electrolyzer at the beginning-of-life (BOL) and end-of-life (EOL) after 200-h of durability test at total current density of 150 mA cm^{-2}. Cell voltage: 2.2 V.

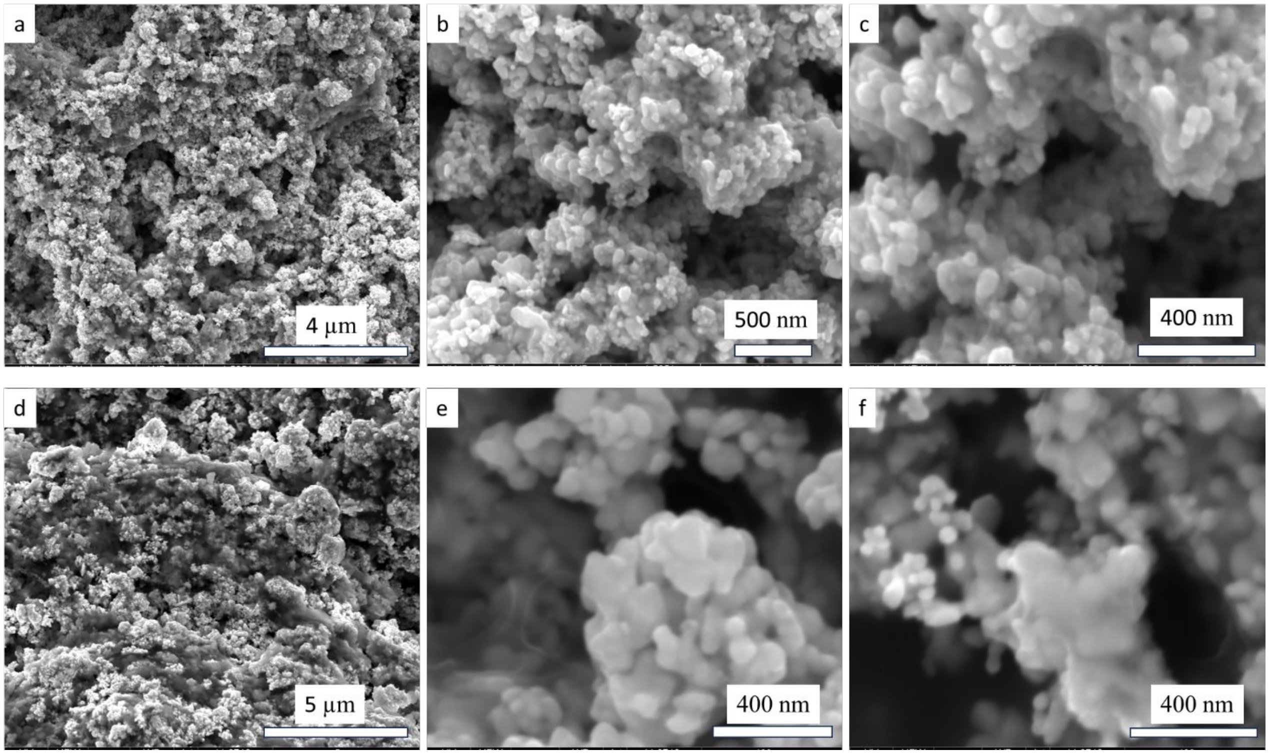

Figure S13. Scanning electron microscopy images of cathode gas diffusion electrode morphology at a)-c) BOL and d)-e) EOL after 200-h of durability test at total current density of 150 mA cm^{-2}.

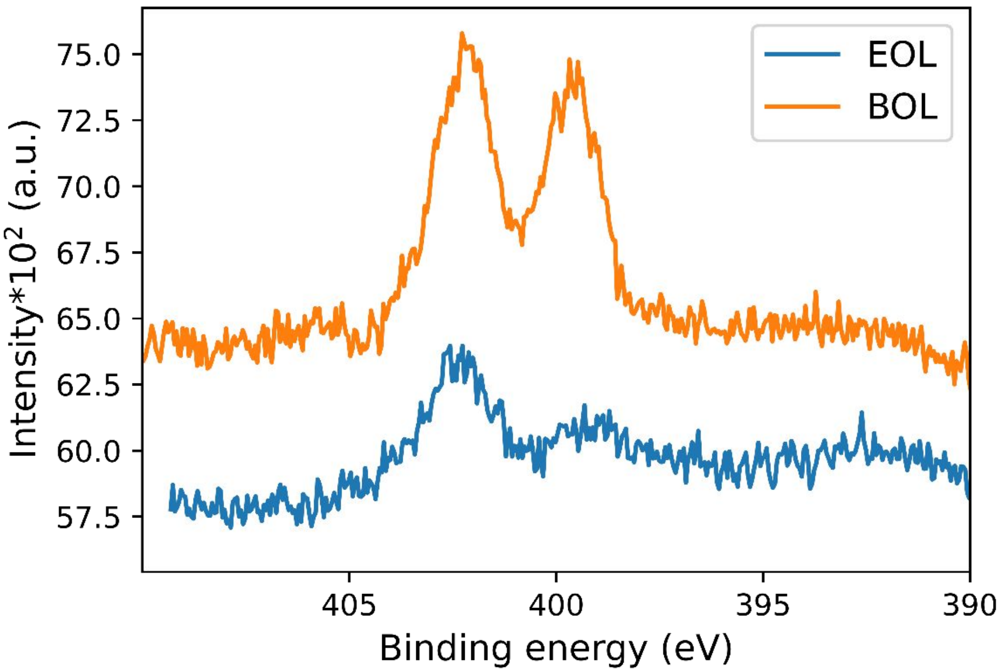

Figure S14. X-ray photoelectron spectra of the N 1s peak for the cathode gas diffusion electrode at BOL and EOL after 200-h of durability test at total current density of 150 mA cm^{-2}.

Table S1. Performance and durability comparison among various water-fed bipolar membrane (BPM) CO_2 electrolyzer for CO production.

CO_2 Electro-lyzer	Anolyte	Cathode polymer electrolyte	Cathode catalyst	Peak CO FE	Peak CO EE	Durability condition (mA cm^{-2})	Average CO-FE in durability test	Durati-on (h)	Starting voltage (V)	Decay rate (mV h^{-1})	Ref
BPM	Water	PiperION	Ag	96%	39%	100	--	200	3.1	0.5	1
BPM	Water	Sustainion	Ag	~85 %	--	--	--	--	--	--	2
BPM	Water	Fumion	Au	<20 %	--	50	--	24	-1.2 vs. RHE	8.3	3
BPM	Water	PiperION	Ag	~95 %	50%	150	79.51%	200	2.65	0.61	This work
BPM	Water	PiperION	Ag	~95 %	50%	300	73.45%	100	2.85	2.1	This work

The average CO FE is calculated by averaging all recorded CO FE during the durability test.

Reference:

1. Disch, J., Ingenhoven, S. & Vierrath, S. Bipolar Membrane with Porous Anion Exchange Layer for Efficient and Long-Term Stable Electrochemical Reduction of CO2 to CO. *Adv. Energy Mater.* **2301614**, 1–9 (2023).

2. O'Brien, C. P. *et al.* Single Pass CO2 Conversion Exceeding 85% in the Electrosynthesis of Multicarbon Products via Local CO2 Regeneration. *ACS Energy Lett.* **6**, 2952–2959 (2021).

3. Pătru, A., Binninger, T., Pribyl, B. & Schmidt, T. J. Design Principles of Bipolar Electrochemical Co-Electrolysis Cells for Efficient Reduction of Carbon Dioxide from Gas Phase at Low Temperature. *J. Electrochem. Soc.* **166**, F34–F43 (2019).
